# JC Polyomavirus Infection of Primary Human Renal Epithelial Cells Is Controlled by a Type I IFN-Induced Response

**DOI:** 10.1128/mBio.00903-16

**Published:** 2016-07-05

**Authors:** Benedetta Assetta, Marco De Cecco, Bethany O’Hara, Walter J. Atwood

**Affiliations:** a Graduate Program in Pathobiology, Brown University, Providence, Rhode Island, USA; b Department of Molecular Biology, Cell Biology, and Biochemistry, Brown University, Providence, Rhode Island, USA

## Abstract

The JC and BK human polyomaviruses (JCPyV and BKPyV, respectively) establish lifelong persistent infections in the kidney. In immunosuppressed individuals, JCPyV causes progressive multifocal leukoencephalopathy (PML), a fatal neurodegenerative disease, and BKPyV causes polyomavirus-associated nephropathy (PVN). In this study, we compared JCPyV and BKPyV infections in primary human renal proximal tubule epithelial (HRPTE) cells. JCPyV established a persistent infection, but BKPyV killed the cells in 15 days. To identify the cellular factors responsible for controlling JCPyV infection and promoting viral persistence, we profiled the transcriptomes of JCPyV- and BKPyV-infected cells at several time points postinfection. We found that infection with both viruses induced interferon production but that interferon-stimulated genes (ISGs) were only activated in the JCPyV-infected cells. Phosphorylated STAT1 and IRF9, which are responsible for inducing ISGs, translocated to the nucleus of JCPyV-infected cells but did not in BKPyV-infected cells. In BKPyV-infected cells, two critical suppressors of cytokine signaling, SOCS3 and SOCS1, were induced. Infection with BKPyV but not JCPyV caused reorganization of PML bodies that are associated with inactivating antiviral responses. Blockade of the interferon receptor and neutralization of soluble interferon alpha (IFN-α) and IFN-β partially alleviated the block to JCPyV infection, leading to enhanced infectivity. Our results show that a type I IFN response contributes to the establishment of persistent infection by JCPyV in HRPTE cells.

## IMPORTANCE

The human polyomaviruses JCPyV and BKPyV both establish lifelong persistent infection in the kidneys. In immunosuppressed patients, BKPyV causes significant pathology in the kidney, but JCPyV is only rarely associated with disease in this organ. The reasons behind this striking difference in kidney pathology are unknown. In this study, we show that infection of primary human renal tubule epithelial cells with JCPyV and BKPyV results in divergent innate immune responses that control JCPyV but fail to control BKPyV. This is the first study that directly compares JCPyV and BKPyV infection *in vitro* in the same cell type they naturally infect, and the significant differences that have been uncovered could in part explain the distinct disease outcomes.

## INTRODUCTION

JC and BK polyomaviruses (JCPyV and BKPyV, respectively) are members of the human *Polyomaviridae* family. JCPyV and BKPyV were isolated in 1971, but 11 additional human polyomaviruses have been discovered in the last decade (1–12). JCPyV is the etiological agent of progressive multifocal leukoencephalopathy (PML), a fatal neurodegenerative disease, and BKPyV causes polyomavirus-associated nephropathy (PyVAN) and hemorrhagic cystitis (HC) (1, 13). JCPyV and BKPyV are common human pathogens, for which 50 to 60% and 80% of healthy individuals, respectively, are seropositive (14–16). Primary infection with JCPyV and BKPyV occurs early during childhood, and it is most often asymptomatic unless there is a preexisting, immunosuppressive condition (17, 18). JCPyV and BKPyV both establish lifelong persistent infections in the kidneys. JCPyV and BKPyV are shed in the urine of 20% and 7%, respectively, of healthy subjects, and viral proteins have been found in renal tubule epithelial cells (14, 19–26).

The mechanism by which JCPyV establishes a persistent infection in the kidney is poorly understood. Only 20% of healthy individuals shed the virus in the urine, while seropositivity rates are 50 to 60% (14). In immunosuppressed adults, JCPyV can traffic from sites of persistence to the central nervous system (CNS), where it causes the destruction of oligodendrocytes, ultimately leading to PML (1, 27, 28). The incidence of PML is about 3 to 5% in individuals with HIV/AIDS (29). Additionally, PML has been reported in patients undergoing immunomodulatory therapies for immune-mediated diseases such as multiple sclerosis (30–32). There are no specific treatments for this rapidly fatal disease.

In contrast, upon immunosuppression BKPyV replicates vigorously in the reno-urinary tract, giving rise to PyVAN in kidney transplant recipients and to hemorrhagic cystitis (HC) in bone marrow transplant patients (12, 13). PyVAN can cause graft dysfunction and premature graft loss in >50% of cases where BKPyV is actively replicating in the organ (33–35). Although JCPyV also persists in the kidney, few cases of nephropathy have been attributed to the virus during immunosuppression (18, 24, 36, 37). Recently, in a cohort of 100 kidney transplant recipients, JCPyV-associated nephropathy was reported to be as low as 0.9%, and overall most diagnosed individuals have normal renal function with no subsequent graft loss (38, 39). Overall, these findings suggest that JCPyV-associated nephropathy is less severe and is associated with a better prognosis. The reasons behind the striking differences between JCPyV- and BKPyV-induced nephropathy are unknown.

JCPyV and BKPyV exist in nature in different variants that can be classified by the sequence of the noncoding control region (NCCR) and by coding region polymorphisms (40–43). Based on their NCCR sequence, viral variants of JCPyV and BKPyV are referred to as archetype and rearranged forms (29, 42). The transmitted form of JCPyV and BKPyV is believed to be the archetype variant because it is the most prevalent form of the virus isolated from the urine of healthy individuals and from sewage waters (42, 44). Less often, viral variants with different levels of rearrangements of the NCCR have been isolated from urine samples of healthy individuals: therefore, it cannot be excluded that these forms are also transmitted (14, 43, 45, 46). It has been hypothesized that the rearranged variants are derived from the archetype isolate during the lifelong infection of the host at the sites of persistence (29, 47, 48). The rearranged variants have been shown to have a replicative advantage *in vitro* over the non-rearranged archetype, and most *in vitro* studies have been carried out using rearranged forms of JCPyV or BKPyV (45, 49, 50). The JCPyV archetype variant does not replicate in human primary kidney cells, and archetype BKPyV produces undetectable levels of large T antigen (TAg) and very little, if any, viral DNA replication in the same cells (51–53). While JCPyV viral variants isolated from PML brains have profound rearrangements in the NCCR, data regarding the association between BKPyV rearranged variants and disease is not as well defined (29). Both archetype and rearranged forms of BKPyV have been isolated from biopsy specimens of kidneys with BKPyV-associated nephropathy or HC (43, 54, 55).

Immune surveillance is important for controlling JCPyV or BKPyV infection in healthy individuals, as immunosuppression places individuals at risk for PML or PyVAN/HC. However, the mechanism by which the immune system controls human polyomaviruses at their sites of persistence is not well described. The innate immune system is the primary line of defense against microbial pathogens, and it is also necessary to prompt an efficient adaptive immune response. Interferons (IFNs) are the primary antiviral cytokines, and they play an important role in the control of RNA and DNA viruses (56–59). IFNs are divided into three families, types I, II, and III, of which the first two are the most studied. The type I IFN family includes IFN-α and IFN-β, and they can be produced by immune and nonimmune cells, while IFN type II, which consists of IFN-γ, is predominantly expressed by natural killer (NK) cells and T cells (60). IFN-α and IFN-β production occurs in response to the activation of pattern recognition receptors (PRRs) by microbial products, and when they are released from cells, they bind to the interferon alpha receptor (IFNAR) on infected cells or uninfected cells (57). This interaction activates a signaling cascade leading to the phosphorylation of signal transducer and activator of transcription (STAT) molecules, which form multimeric complexes and translocate to the nucleus to induce interferon-stimulated genes (ISGs) (61). ISGs block the viral life cycle at different stages, including viral entry, replication, assembly, and egress (59, 62). Previous studies have shown that mouse embryonic fibroblasts (MEFs) stably expressing simian virus 40 (SV40), JCPyV, or BKPyV large T antigens (TAg) in the absence of viral infection induce ISGs and that the antiviral state requires STAT1 (63, 64). The antiviral effect of IFN-α and IFN-β made ISGs likely candidates for controlling the mechanism of JCPyV persistence and inhibition of JCPyV-induced nephropathy.

The goal of our study was to directly compare JCPyV and BKPyV infections in primary human renal epithelial cells and to investigate specific mechanisms that could favor persistence or disease. We found that JCPyV infection of primary human renal epithelial cells remained constant and low over 3 weeks, while BKPyV spread efficiently, killing the cells by day 15. We used next-generation sequencing (NGS) to compare biological pathways differentially activated in JCPyV- versus BKPyV-infected cells and found that JCPyV significantly induced ISGs. Phosphorylated STAT1 (pSTAT1) and interferon regulatory factor 9 (IRF9) were found in the nucleus of JCPyV-infected cells, and the block of interferon alpha/beta receptor signaling partially increased JCPyV infectivity. The marked difference in cell responses between the two viruses was not due the inability of BKPyV to stimulate IFN production. In fact, both JCPyV- and BKPyV-infected cells released IFN-β, but BKPyV-infected cells did not activate the antiviral response mediated by the cytokine, as confirmed by the lack of pSTAT1 in the nucleus of BKPyV-infected cells. It is likely that BKPyV actively interferes with the signaling cascade mediated by type I interferon. Our data showing that BKPyV but not JCPyV causes a fundamental rearrangement of PML nuclear bodies (NBs) support this hypothesis.

## RESULTS

### JCPyV establishes a low-level persistent infection in HRPTE cells.

**FIG 1:**
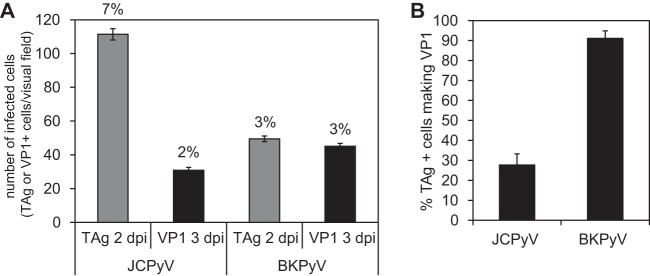
JCPyV productively infects HRPTE cells. HRPTE cells were infected with JCPyV or BKPyV, fixed at 2 or 3 days postinfection (dpi), and stained for TAg or VP1, respectively. Cells were visualized by fluorescence microscopy, and the number of positive nuclei was assessed at ×10 magnification (A). The total number of cells per visual field was determined using DAPI nuclear staining, and the percentage of TAg- or VP1-positive cells is reported on top of each bar in panel A. TAg-positive nuclei were set to 100%, and the percentage of VP1-positive nuclei was consequently calculated based on the results presented in panel A (B). Results represent the average of three independent experiments in triplicates, and error bars represent standard errors (SE).

**FIG 2:**
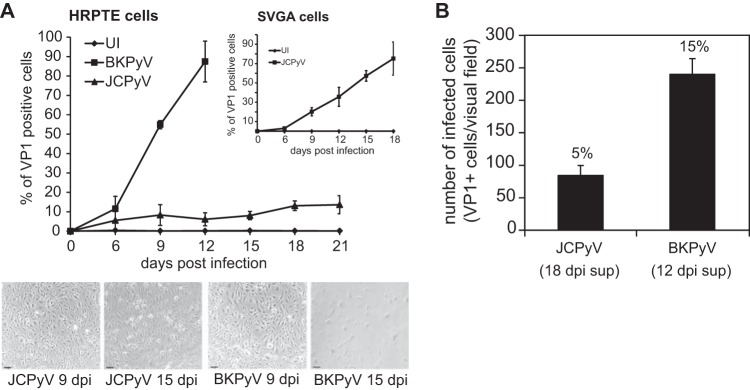
JCPyV establishes a low-level persistent infection in HRPTE cells. HRPTE cells were infected with JCPyV or BKPyV, and expression of VP1 was assessed every 3 days for 21 days by flow cytometry. Results are expressed as percentages of VP1-positive cells calculated using FlowJo (A). SVGA cells were infected with JCPyV and stained as previously stated (inset). Supernatants from infected HRPTE cells were collected at 18 dpi and 12 dpi for JCPyV and BKPyV, respectively, and used to infect newly plated HRPTE cells. Cells were fixed and stained for VP1 at 3 dpi, and positive nuclei were counted using fluorescence microscopy at ×10 magnification (B). The total number of cells per visual field was determined using DAPI nuclear staining, and the percentage of VP1-positive cells is reported on top of each bar in panel B. Results represent the average from three independent experiments, and error bars represent standard deviations (SD). In panel A, a total of 30,000 events were recorded per time point.

Human renal proximal tubule epithelial (HRPTE) cells were challenged with JCPyV and BKPyV, and the infection was monitored by immunofluorescence staining of large T antigen (TAg) and major structural protein VP1. At 2 days postinfection (dpi), more cells expressed JCPyV TAg than BKPyV TAg, but by 3 dpi, the numbers of JCPyV and BKPyV VP1-expressing cells were similar (Fig. 1A). When expressed as the percentage of TAg-positive cells that went on to make VP1, almost 100% of the BKPyV-infected cells also made VP1, while a much smaller percentage of JCPyV-infected cells progressed to late viral protein production (Fig. 1B). As infection progressed, we observed that BKPyV killed all of the cells by day 15, while JCPyV maintained a low-level persistent infection over the course of the experiment (Fig. 2A). This was specific to HRPTE cells as JCPyV at the same multiplicity of infection (MOI) grew vigorously in human glial cells (SVGA) (Fig. 2A, inset). As expected, the amount of BKPyV released into the cell supernatant at 12 dpi was significantly larger than the amount of JCPyV released at 18 dpi (Fig. 2B). Infection of HRPTE cells with a higher JCPyV MOI did not affect the persistent phenotype observed (data not shown).

### ISGs are upregulated in JCPyV-infected HRPTE cells compared to BKPyV-infected cells starting at 6 days postinfection.

**FIG 3:**
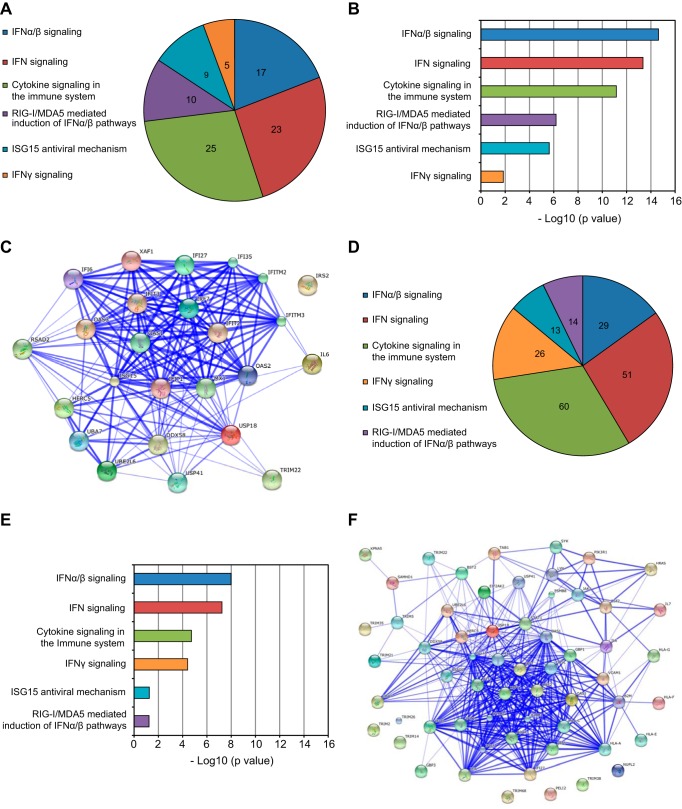
Interferon-stimulated genes are upregulated in JCPyV-infected HRPTE cells compared to BKPyV-infected cells starting at 6 days postinfection. HRPTE cells were infected with either JCPyV or BKPyV, and at 3, 6, and 9 dpi, total RNA was harvested and RNA-seq and bioinformatics analyses were performed. Genes from JCPyV-infected cells were compared with those from BKPyV-infected cells. The lists of genes were uploaded to a gene enrichment analysis tool called Enrichr. Pie charts of the top 6 biological pathways were generated with the number of genes assigned by Enrichr to each pathway at 6 dpi (A) and at 9 dpi (D). Enrichr provided *P* values using Fisher’s exact test, and −log_10_ (*P* values) are displayed for the top 6 biological pathways at 6 dpi (B) and 9 dpi (E). The interactions among genes that were assigned to the biological pathway “cytokine signaling in the immune system” were visualized using STRING10 at 6 dpi (C) and at 9 dpi (F); stronger associations are represented by thicker lines.

To begin to understand the host cell factors involved in restricting JCPyV infection, we profiled the transcriptomes of JCPyV- and BKPyV-infected HRPTE cells as well as uninfected cells at 3, 6, and 9 days postinfection. We first compared JCPyV- to BKPyV-infected samples and identified a total of 108 genes that were differentially expressed on day 3 postinfection, 623 genes that were differentially expressed on day 6, and 8,350 genes that were differentially expressed on day 9. In the JCPyV-infected cells, we recorded an exponential increase in the number of interferon-stimulated genes: 11 genes at 3 dpi, 142 at 6 dpi, and 658 at 9 dpi (obtained using the Interferome tool). The list of differentially expressed genes at each time point was further analyzed using a web-based tool called “Enrichr” that provides visualization summaries of biological functions from the gene lists provided. We concentrated our analysis on biological pathways detected by the Reactome tool set. At 6 and 9 dpi, the most significantly enriched pathways were related to the innate immune system and to an interferon response (ISGs) (Fig. 3). In JCPyV-infected cells at 6 dpi, 17 genes were assigned to the “interferon alpha/beta signaling” pathway, 23 genes to the “interferon signaling” pathway, and 25 to the “cytokine in the immune system” pathway (Fig. 3A). By 9 dpi, the number of genes assigned to each group was significantly increased (Fig. 3D). Enrichr uses the Fisher exact test to calculate the statistical significance of overlap between the input list and the gene sets in the Reactome library. We plotted the −log_10_ value (*P* value) calculated for each pathway at 6 and 9 dpi (Fig. 3B and E). Additionally, we used STRING to visualize known and predicted protein interactions among the genes included in the “cytokine signaling in the immune system” group at 6 and 9 dpi (Fig. 3C and F). BKPyV-infected cells showed a significant overexpression of IFN-α and IFN-β subtypes as well as IFN-γ at 9 dpi. *SOCS3* and *SOCS1* were upregulated at 9 dpi, with SOCS3 being significantly overexpressed also at 6 dpi in the BKPyV-infected cells. SOCS1 and -3 are well-known suppressors of cytokine signaling, and among other functions, they block the induction of ISGs (65). ISG overexpression in BKPyV-infected cells compared to uninfected cells was limited to a single gene (*HERC5*) at 9 dpi and to four genes at 6 dpi (*IFI6*, *IRF7*, *OAS3*, and *HERC5*) (see Table S1 in the supplemental material). These genes were also overexpressed in JCPyV-infected cells, but to a much greater extent. Interestingly, we found *IRF4* upregulated at 6 and 9 dpi and *MX2* only at 9 dpi. These two genes were uniquely found in BKPyV-infected cells. When we analyzed genes downregulated in BKPyV-infected cells compared to uninfected cells, we detected *IL6*, *IFI16*, and *DTX4* both 6 and 9 days postinfection. Additionally, we found *STAT1*, *JAK2*, *PML*, *IFITM2* and -*3*, *ADAR*, and *IFIT2* to be downregulated at 9 days postinfection. Our results showed that JCPyV- but not BKPyV-infected cells are robustly inducing ISGs starting at day 6 postinfection. Importantly BKPyV, but not JCPyV, induces *SOCS3* and *SOCS1* and downregulates genes involved in the IFN signaling cascade and ISGs. Furthermore, genes involved in the regulation of the cell cycle were the most significantly upregulated set in both JCPyV- and BKPyV-infected versus uninfected cells (see Fig. S1 in the supplemental material). These results confirm the known ability of polyomaviruses to induce DNA replication and cell growth to successfully complete the viral life cycle (66).

### JCPyV- and BKPyV-infected cells produce IFN-β, but JCPyV is more sensitive to the cytokine.

**FIG 4:**
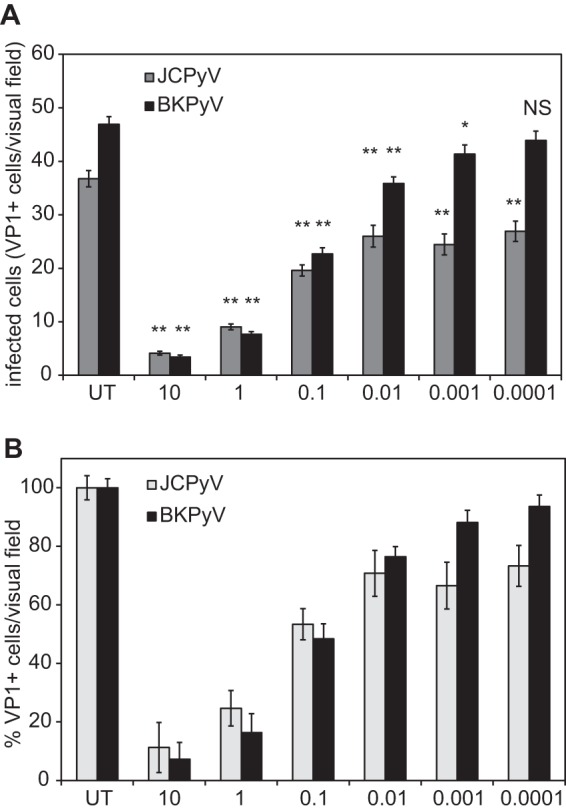
JCPyV infection is more sensitive than BKPyV to low concentrations of IFN-β. HPRTE cells were pretreated with decreasing concentrations of IFN-β, expressed as international units (IU), for 6 h. Cells were then infected with JCPyV or BKPyV, fixed and stained for VP1 at 3 dpi. Samples were visualized by fluorescence microscopy, and results are expressed as number of VP1-positive nuclei per visual field at ×10 magnification (A) or percentage of VP1-positive nuclei per visual field when untreated cells (UT) were set to 100% (B). Results represent the average from three independent experiments in triplicates, and error bars represent standard errors (SE). **, *P* < 0.01; *, *P* < 0.05.

To quantify IFN-β production, we infected HRPTE cells with JCPyV and BKPyV and performed enzyme-linked immunosorbent assays (ELISAs) on the supernatants collected at 3, 6, and 9 dpi (Table 1). In supernatants of JCPyV-infected cells, significant amounts of cytokine were detected at 6 and 9 dpi but not at the earlier 3-dpi time point. IFN-β was detected in the media from BKPyV-infected cells at all three time points. No significant change in expression was detected between 24 and 48 h postinfection in either BKPyV- or JCPyV-infected cells when tested by quantitative PCR (qPCR) (data not shown). To determine whether pretreatment of cells with small amounts of IFN-β could reduce JCPyV or BKPyV infection, HRPTE cells were pretreated with decreasing concentrations of IFN-β for 6 h, infected with JCPyV or BKPyV, and stained for VP1 at 3 dpi (Fig. 4A and B). Both JCPyV and BKPyV were equally sensitive to concentrations of IFN-β ranging from 10 to 0.1 IU. JCPyV remained sensitive to lower concentrations of the cytokine, resulting in a constant ~30% decrease in VP1 expression at 0.01, 0.001, and 0.0001 IU. BKPyV, however, became progressively less sensitive to the antiviral activity of IFN-β, and at 0.0001 IU, there was no difference between treated and untreated cells. Our data show that JCPyV induces IFN-β starting at 6 days postinfection, while BKPyV induces it starting at 3 days postinfection. Additionally, JCPyV is more sensitive to low physiologically relevant concentrations of type I IFN than BKPyV.

**TABLE 1:** JCPyV- and BKPyV-infected HRPTE cells produce IFN-β^a^

Cells	IFN-β concn in pg/ml (SE) at:		
	3 dpi	6 dpi	9 dpi
Uninfected	<LOD (0)	<LOD (0)	<LOD (0)
JCPyV infected	<LOD (0)	54.94 (0.97)	57.23 (2.39)
BKPyV infected	60.09 (2.22)	53.02 (0.78)	67.87 (4.99)

a HRPTE cells were infected with JCPyV and BKPyV, supernatants were harvested at 3, 6, and 9 dpi, and the IFN-β concentration was determined by ELISA. Results represent the average from three independent experiments, and the standard error (SE) is indicated in parentheses. LOD, limit of detection.

### pSTAT1 and IRF9 colocalize in JCPyV-infected nuclei.

**FIG 5:**
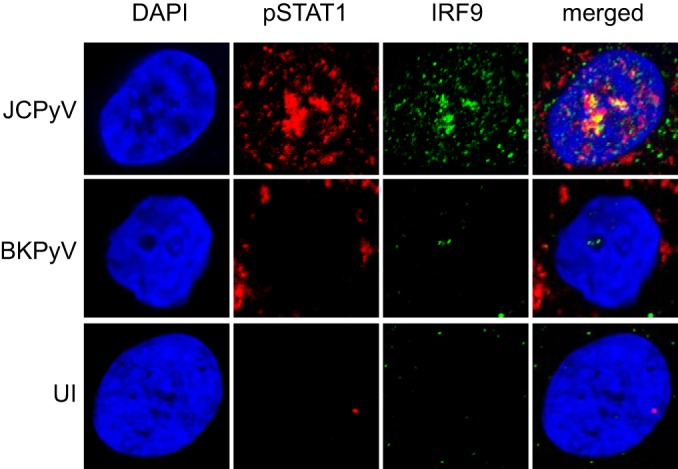
pSTAT1 and IRF9 colocalize in the nucleus of JCPyV-infected HRPTE cells. HRPTE cells were infected with JCPyV or BKPyV, fixed at 8 dpi, and stained for pSTAT1 (red) and IRF9 (green) using indirect immunofluorescence. Nuclei were visualized by DAPI staining. Uninfected cells (UI) were used as a control. Samples were imaged by confocal microscopy at ×63 magnification. The panel shows representative images from two independent experiments.

**FIG 6:**
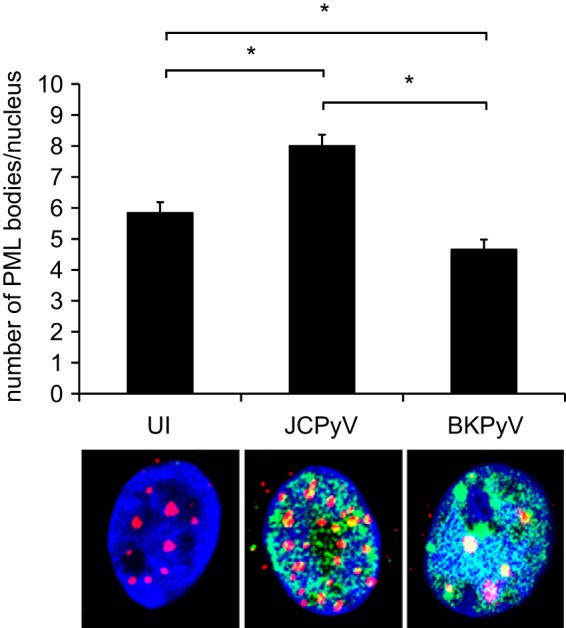
The number of PML bodies per nucleus is significantly increased in JCPyV-infected cells. HPRTE cells were infected with JCPyV or BKPyV, fixed at 8 dpi, and stained for PML (red) and TAg (green) using indirect immunofluorescence. Nuclei were visualized by DAPI staining. Uninfected cells (UI) were used as a control. Samples were imaged by confocal microscopy, and at least 120 cells per replicate were recorded for a total of 360 to 450 cells. Images were processed using Cell Profiler, and the average number of PML bodies per nucleus was calculated. Error bars represent standard errors (SE). *, *P* < 0.05.

We infected HRPTE cells with JCPyV and BKPyV and stained for pSTAT1 and IRF9 to determine the nuclear translocation of the protein complex, one of the crucial steps in ISG induction (Fig. 5). JCPyV-infected nuclei showed positive colocalization signal for pSTAT1 and IRF9. In contrast, BKPyV-infected cells showed low signal for IRF9 and low to no signal for pSTAT1 in the nucleus. Furthermore, we quantified PML nuclear bodies (NBs) per nucleus in JCPyV- and BKPyV-infected cells. PML NBs possess interferon (IFN)-mediated antiviral effects, and a link between PML NB reorganization and BKPyV infection has been described previously (67) (Fig. 6). JCPyV caused a significant increase in the total amount of PML NBs per nucleus, consistent with the induction of the antiviral response. Additionally, we confirmed previous findings as BKPyV was able to induce significant PML NB reorganization at 8 dpi.

### Blockade of type I IFN-mediated signaling increases JCPyV infection.

**FIG 7:**
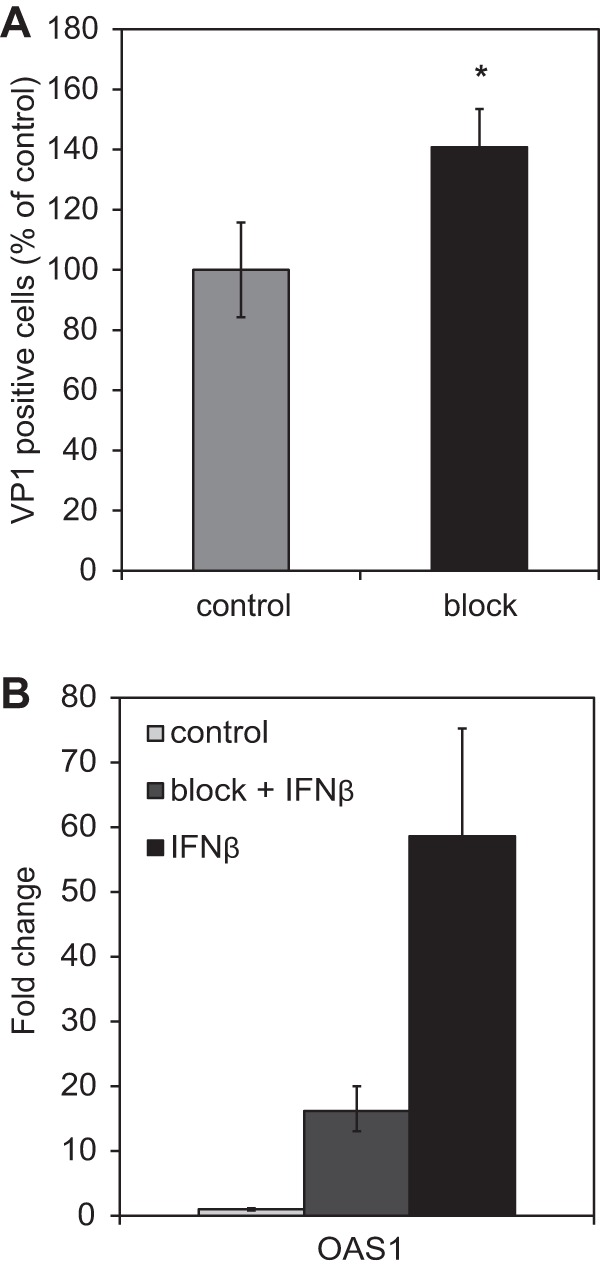
Blockade of type I IFN-mediated signaling increases JCPyV infection. HRPTE cells were infected with JCPyV. At day 4 postinfection, cells were treated with anti-IFNAR2 blocking antibody for 1 h, and then medium containing neutralizing anti-IFN-β and anti-IFN-α was added on top. The treatment was repeated every 2 days, infection levels were assessed at 11 dpi by VP1 staining, and positive cells were quantified using flow cytometry. Percentages of positive cells were calculated using FlowJo. The increased percentage of infection in treated cells was calculated by setting the untreated cells to 100% (A). The results represent the average of two independent experiments performed in duplicate for a total of 40,000 events per condition. To confirm the efficacy of our approach, HRPTE cells were treated with anti-IFNAR2 blocking antibody and with 0.1 IU of IFN-β. Total RNA was harvested, and qPCR was performed using *OAS1* targeting primers (B). The results represent the average from two independent experiments. *, *P* < 0.05.

To determine whether the restriction of JCPyV infection was indeed due to IFN stimulation of ISGs, we blocked the interferon alpha/beta receptor and neutralized IFN-α and IFN-β released from the infected cells using blocking antibodies. This block resulted in an increase in JCPyV-infected cells (Fig. 7A). In control experiments, the same treatment blocked expression of a known ISG (*OAS1*) (Fig. 7B).

## DISCUSSION

JCPyV and BKPyV persist in the kidney of healthy individuals; however, upon immunosuppression, they cause different diseases in different organs. BKPyV causes severe disease in the kidney (PyVAN and HC), while JCPyV causes fatal neurodegeneration in the brain (PML), with little to no effect on the kidney. The goal of our study was to investigate JCPyV and BKPyV infection in primary human renal epithelial cells to begin to understand the molecular basis of such dissimilar outcomes. Our results showed that over the course of 21 days, JCPyV established a persistent infection in primary human renal epithelial cells, while BKPyV lysed all of the cells by day 15. JCPyV and BKPyV were able to induce IFN-β production, but ISGs were only detected in JCPyV-infected cells starting at day 6 post-initial infection. Furthermore, blockade of the interferon alpha/beta receptor signaling activation partially enhanced JCPyV infectivity. Our data show that a type I IFN response contributes to the establishment of persistent infection by JCPyV in primary human renal epithelial cells. Our results also suggest that BKPyV blocks the downstream activation of ISGs mediated by IFN.

The innate immune system utilizes receptors called pattern recognition receptors (PRRs) to recognize microbial products. PRRs can be found on the membranes of cells or intracellular organelles, as well as in the cytosol or nucleus of immune and nonimmune cells (68–71). PRRs have not yet been identified for polyomaviruses. Interestingly, it has been recently reported that ectopic expression of SV40 TAg induces a DNA damage response that activates IRF1, causing IFN-β production and subsequent ISG expression (72). Many of these sensors are responsible for the induction of IFN that results in the activation of the classical “antiviral state” and is also crucial to stimulate an optimal adaptive immune response (71). When type I IFNs are released, they bind to the IFNAR, which activate janus kinase 1 (JAK1) and tyrosine kinase 2 (TYK2): in turn, they phosphorylate STAT1 and STAT2 which dimerize, assemble with IRF9, and are translocated in the nucleus, where they induce ISG expression (61). These effector molecules are able to block the viral life cycle at different stages to mount a diverse and redundant antiviral response (59, 62). Furthermore, it has been shown that expression of such genes can also have additive effects on viral infection (73, 74). The specific activity of ISGs on the JCPyV life cycle has never been explored. In the present study, we demonstrated nuclear translocation of pSTAT1 and IRF9 in JCPyV-infected cells and identified multiple ISGs robustly upregulated, including *IRF7*, *ISG15*, *MX1*, *IFI6*, *RASD2* (viperin), *OAS1* to -*3*, and *IFITM2* and -*3* (Fig. 3 and 5). *IRF7* is a member of the interferon regulatory factor family, it is strongly induced by type I IFN-mediated signaling, and it regulates the transcription of type I IFN genes (75). The other genes mentioned have a broad activity against different RNA and DNA viruses. *ISG15*, for example, has been reported to directly inhibit virus release and to target viral or host proteins for ISGylation, a process similar to ubiquitination (59, 76–80). *STAT1* is widely expressed in the cytoplasm of cells and can be phosphorylated to induce ISG expression. *STAT1* is also an interferon-stimulated gene itself, so the antiviral response can be amplified (61). Additionally, by blocking IFN-mediated signaling, we partially increased VP1 expression, confirming that the type I IFN-mediated response in fact reduced JCPyV infectivity. This response appears to be activated late in infection as neither IFN-β nor ISGs are detected at 3 dpi despite JCPyV genome replication (Fig. 1). Interestingly, when we compared the amounts of JCPyV TAg and VP1 at 2 and 3 dpi, we noticed that JCPyV VP1 expression did not correlate with the abundant expression of TAg. This may indicate that the persistent phenotype we see in HRPTE cells is the result of a cell-intrinsic restriction acting on JCPyV replication early during infection and that the IFN-mediated antiviral response is contributing to keeping the level of replication low over time. To support our hypothesis, we blocked IFN-mediated signaling at 4 and 6 dpi and stained for VP1, respectively, at 6 and 8 dpi, and we did not see an increase in VP1-positive cells (data not shown). We could not find overexpression of relevant transcription factors known to have an inhibitory role against JCPyV replication, leaving us unable to determine how the first round of JCPyV replication is controlled. The inhibitory activity of IFN-β on JCPyV infection has been previously reported in primary or immortalized human glial cells (81, 82). However, the lowest concentration that has been used in those studies was 5 IU (82). In the present study, we tested concentrations of IFN-β down to 0.0001 IU because after our ELISA quantification, we reasoned that the amount of cytokine produced in HRPTE cells would be more in the range of 10 IU and below. We demonstrated that even as little as 0.0001 IU of IFN-β was enough to mount an antiviral response against JCPyV infection, which was reduced by approximately 30% (Fig. 4B).

On the other hand, we observed that BKPyV is highly cytopathic in HRPTE cells, killing all cells by day 15 despite a low MOI. The amount of TAg- and VP1-expressing cells correlate: in fact, roughly 90% of cells expressing TAg went on to make VP1, indicating that HRPTE cells do not restrict BKPyV replication. As ISGs were significantly upregulated in JCPyV compared to BKPyV, we further investigated the overexpressed genes in BKPyV-infected cells compared to uninfected cells. We detected IFN-β production at 3 dpi, but no ISGs were detected at this time point. Interestingly, IRF7 was slightly upregulated at 6 dpi (see Table S1 in the supplemental material). This may indicate that a small activation of the signaling cascade took place in BKPyV-infected cells as a consequence of the production of IFN-β. At 6 and 9 dpi, we also detected the upregulation of SOCS3, a suppressor of cytokine signaling. SOCS3 can be stimulated by JAK/STAT signaling mediating a feedback inhibition or can be induced via infection by several viruses, including herpes simplex virus 1 (HSV-1), influenza A virus (IAV), and HIV (83–86). Importantly, SOCS3 induction by the above-mentioned viruses always leads to inhibition of type I IFN signaling, thus enhancing viral replication (83–86). Based on our results, we cannot determine exactly whether SOCS3 is stimulated by JAK/STAT signaling or directly by BKPyV infection. However, it is reasonable to hypothesize that SOCS3 inhibited the IFN-mediated signaling activation blocking the induction of ISGs. This is consistent with the fact that *IRF7* was no longer overexpressed at 9 dpi, that pSTAT1 was not detected in the nucleus of BKPyV-infected cells, and that several ISGs were actually downregulated compared to those in uninfected cells.

Promyelocytic leukemia nuclear bodies (PML NBs), also known as nuclear domain 10 (ND10), are dynamic, small, punctate subnuclear structures with a diverse array of functions, including transcription regulation, DNA damage response, and chromatin remodeling. Interestingly, they possess both intrinsic and interferon (IFN)-mediated antiviral effects against several DNA and RNA viruses (87, 88). Previously published data from our laboratory show that pretreatment of SVGA cells with IFN-β causes a significant increase in the amount of PML NBs per nucleus, and as a consequence, infection was inhibited (89). Interestingly, when PML bodies were disrupted by arsenite treatment and the cells subsequently supplemented with IFN-β, PML NBs did not reform, and infection was no longer inhibited, suggesting that IFN-β was blocking infection of SVGA cells through the help of PML NBs (89). In the present study, we found that the number of PML bodies per nucleus in HRPTE cells infected with JCPyV was significantly increased at 8 days postinfection, consistent with the timing of the antiviral response activation (Fig. 6). One intriguing hypothesis is that the intrinsic antiviral activity of PML NBs is responsible for partially restricting JCPyV replication early during infection, similar to what has been reported for HPV (87). Then, after the IFN signaling cascade is activated, the number of PML NBs increases, and in concert with other ISGs, viral replication is kept low over time. Interestingly, Jiang et al. showed that BKPyV infection of HRPTE cells causes a functional reorganization of PML NBs (67). The total number of PML NBs per nucleus was decreased, and the average size was instead increased, resulting in the inactivation of their antiviral activity (67). In fact, the growth of an ICP0-null mutant HSV-1 strain was rescued by preinfection with BKPyV (67). In the present study, we recapitulated their findings and saw a significant decrease in the number of PML NBs per nucleus at 8 dpi (Fig. 6). Overall we can conclude that human primary kidney epithelial cells have multiple ways to restrict viral infection, and it appears that such mechanisms are successful at controlling JCPyV infection. Conversely, the same mechanisms are not enough to control BKPyV as it appears to have evolved multiple ways to counteract the restrictions human primary kidney epithelial cells have in place.

## MATERIALS AND METHODS

### Cells and viruses.

Primary renal proximal tubule epithelial cells were obtained from ATCC (American Type Culture Collection) and were grown in renal epithelial cell basal medium (RECBM) supplemented with one Renal epithelial cell growth kit as suggested by the manufacturer (ATCC). For JCPyV infection, we used a lab-adapted strain referred to as Mad-1/SVEΔ, which was described previously (90, 91). For BKPyV infection, we used the Dunlop strain purchased from ATCC. JCPyV and BKPyV were grown in SVGA cells (human glial cells transformed with SV40 large T antigen) and Vero cells, respectively, using 1,700-cm^2^ roller bottles. Cells were cultured for 14 days, with the cell culture medium replaced at 7 days. Viral lysates were harvested by scraping cells in the presence of cell culture medium, and this lysate was frozen and thawed 3 times. When needed, JCPyV and BKPyV lysates were purified as previously described (92).

### Indirect immunofluorescence assay of JCPyV and BKPyV infection.

For long-term experiments, scoring of infection was done by flow cytometry. For short-term experiments, cells were stained and counted by eye using epifluorescence microscopy (Nikon E800) as previously described (93). Briefly, plates were fixed using ice-cold 100% methanol at −20°C for at least 20 min. They were treated with phosphate-buffered saline (PBS) containing 1% Triton X-100 for 5 min and blocked with 10% goat serum for 30 min at room temperature (RT). Samples were incubated for 1 h at 37°C with PAB597 (1:50) or AB-2 (1:20) followed by staining with secondary antibody conjugated to Alexa Fluor 488 (1:500). Data were expressed as VP1- or TAg-positive cells/visual field. For flow cytometry quantification, HRPTE cells were washed once with PBS and detached with trypsin (Mediatech, Inc.). The cells were then transferred to V-bottom 96-well plates and pelleted by centrifugation at 600 × *g* for 5 min, washed with PBS, and fixed in 0.2 ml 4% paraformaldehyde (PFA) for 10 min. Cells were pelleted, washed with PBS, and permeabilized with 0.2 ml PBS containing 1% Triton X-100 for 10 min at RT. Cells were then pelleted and resuspended in 0.1 ml PBS containing an Alexa Fluor-labeled purified monoclonal antibody to VP1 (PAB597-AF488 [1:100]). After incubation for 1 h at RT, cells were washed once with PBS, and fluorescence was read by flow cytometry on a FACSCanto II fluorescence-activated cell sorter (BD Biosciences). Uninfected cells were used to establish gates for infected cells, and data were analyzed using FlowJo software (Tree Star, Inc.) and expressed as percentage of VP1-positive cells.

### Time course experiments.

Long-term experiments were performed as follows. HRPTE cells were infected with JCPyV or BKPyV lysates at a multiplicity of infection (MOI) of 0.03 fluorescence-forming unit (FFU)/cell. Virus was diluted in culture medium, and cells were infected for 2 h. Virus was aspirated off, and cells were incubated at 37°C and 5% CO_2_ for 21 days. Cells were fed every 2 days and medium replaced every 6 days. Cells were harvested, and infection was scored by flow cytometry as described above every 3 days starting at 6 days postinfection. Short-term experiments were performed as follows: HRPTE cells were infected as described above, infections lasted for 2 or 3 days, and cells were stained for large T antigen (TAg) at day 2 or VP1 at day 3. For transcriptome profiling and ELISA experiments, purified JCPyV and BKPyV were used at an MOI of 0.03 FFU/cell. Infection was carried out for 9 days, and every 3 days, total RNA was harvested using the RNeasy minikit (Qiagen) with DNase treatment for subsequent transcriptome profiling. At each time point, supernatants were also collected for ELISA. Cells were fed, but medium was never replaced over the 9-day period.

### ELISA, antibodies, and primers.

To quantify IFN-β in cell culture media, we used the VeriKine human interferon beta ELISA kit purchased from PBL Assay Science with a range of detection of between 50 and 4,000 pg/ml (catalog no. 41410-1B). We used the monoclonal antibody PAB597 to detect JCPyV and BKPyV VP1. To detect JCPyV and BKPyV TAg, we used a mouse monoclonal antibody to SV40 large T antigen (AB-2) purchased from Calbiochem at a 1:20 dilution, which cross-reacts with both human polyomaviruses. To detect PML protein, we used a polyclonal antibody purchased from Santa Cruz Biotechnology (sc-5621) at a 1:200 dilution. To block interferon alpha/beta receptor, we used a neutralizing monoclonal antibody directed at chain 2 of the protein at a final concentration of 20 µg/ml (PBL Assay Science catalog no. 21385-1). To neutralize secreted IFN-β and IFN-α, we used two different monoclonal antibodies at a final concentration of 6 µg/ml each (PBL Assay Science catalog no. 21400-1 and 21116-1, respectively). To detect pSTAT1, we used a monoclonal antibody purchased from Santa Cruz Biotechnology (sc-8394) at 1:25 dilution, and to detect IRF9, we used a polyclonal antibody purchased from Novus Biologicals (NBP2-16991) at a 1:100 dilution. To perform qPCR for OAS1, the following primers were used: forward, TGGAGACCCAAAGGGTTGGA, and reverse, AGGAAGCAGGAGGTCTCACC.

### Transcriptome profiling of infected HRPTE cells and data analysis.

Total RNA was harvested as described above, quantification was performed using NanoDrop 2000c, and the quality of the RNA was determined with an Agilent 2100 Bioanalyzer. Library preparation, RNA sequencing (RNA-seq), and bioinformatics analysis were performed by Beckman Coulter Genomics (Genewiz). Libraries were prepared with a TruSeq stranded total RNA sample with a Ribo‐Zero prep kit, and the RNA sequencing read type was 2 × 50 bp. The amount of quality control (QC) passed reads varied between ~60 and 99 million across all samples infected and uninfected. Of these reads, between 90 and 99% of the pairs mapped to the human genome reference sequence GRCh38. The mapping was performed using Tophat version 2.0.10 in conjunction with Bowtie version 1.0.0. Cufflinks 2.1.1 was used to detect genes and transcripts, and then Cuffdiff 2.1.1 was used to collect FPKM expression values (i.e., fragments per kilobase of exons and per million of mapped reads). EdgeR Bioconductor 2.12 package was used to determine differentially expressed genes. We obtained ~60 million reads per sample. Genes from JCPyV-infected cells were compared with those from BKPyV-infected cells at each of the time points tested (3, 6, and 9 dpi). Additionally, JCPyV-infected cells and BKPyV-infected cells were, respectively, compared to uninfected cells. Results were filtered for a log_2_ fold change (FC) of ≥1 or ≤−1, *P* value of ≤0.05, and false discovery rate (FDR) of ≤0.05. The lists of genes were uploaded to two different gene enrichment analysis tools: Interferome (http://www.interferome.org/interferome/home.jspx) and Enrichr (http://amp.pharm.mssm.edu/Enrichr/) (94, 95). Interferome is a database that contains type I, II, and III interferon (IFN)-regulated genes, manually curated from publicly available microarray data sets from cells treated with IFN. This tool has been used to estimate how many ISGs were upregulated at each time point collected for cells infected with JCPyV or BKPyV. The gene set libraries provided by Enrichr are divided into six categories: transcription, pathways, ontologies, diseases/drugs, cell types, and miscellaneous. Enrichr was used to identify specific biological pathways significantly represented in any given gene set. Such biological pathways were obtained from the reactome pathway database (http://www.reactome.org/pages/about/reactome/). Interactions among genes were visualized using STRING (http://string-db.org/newstring_cgi/show_input_page.pl?UserId=xB5_QHMTyV9u&sessionId=mmAKTMv33duO). STRING is a database of known and predicted protein interactions, including direct (physical) and indirect (functional) associations (96).

### Pretreatment of cells with IFN-β and blockade of IFN signaling.

HRPTE cells were plated in 96-well plates and the following day treated for 6 h with different concentrations of IFN-β (PBL Assay Science catalog no. 11415-1.) Ten international units was serially diluted in culture medium down to 0.0001 IU. IFN-β was removed, and cells were infected with JCPyV or BKPyV for 2 h, fixed at 3 dpi, and stained for VP1 as described above. VP1-positive cells were counted using epifluorescence microscopy (Nikon E800). To block IFN signaling, cells were prechilled on ice for 25 min, interferon alpha/beta receptor blocking antibody was added, and the cells were incubated for 1 h on ice. Fresh medium supplemented with a cocktail of anti-IFN-α and anti-IFN-β neutralizing antibodies was added without removing the interferon alpha/beta receptor blocking antibody. The treatment was repeated every 2 days, and cells were stained for VP1 at day 11 using immunofluorescence staining scored by flow cytometry and analyzed using FlowJo (cell analysis software).

### Confocal imaging.

HRPTE cells were plated on coverslips in 24-well plates, and they were infected with JCPyV or BKPyV at a MOI of 0.03 FFU/cell for 2 h. Virus was aspirated off and replaced with fresh medium. Cells were fed with 200 µl of medium every 2 days, and medium was replaced at day 6. At day 8 postinfection, cells were washed with 1× PBS and then fixed with 4% PFA for 10 min at RT. Cells were permeabilized using PBS containing 1% Triton X-100 for 10 min at RT. Samples were blocked with 10% goat serum for 30 min at RT. Cells were costained overnight at 4°C for PML protein and TAg or for pSTAT1 and IRF9. The following day, cells were washed 3 times with 1× PBS, and the staining was revealed by incubation for 1 h at 37°C with a secondary antibody conjugated to Alexa Fluor 633 and 488, respectively. Finally, nuclei were stained with DAPI (4′,6-diamidino-2-phenylindole) and coverslips mounted on slides. Samples were imaged using an LSM-710 laser scanning confocal microscope with a 63× objective (Carl Zeiss). The number of PML NBs per nucleus was calculated using CellProfiler (97).

## SUPPLEMENTAL MATERIAL

Figure S1  Genes involved in the regulation of the cell cycle were the most significantly overexpressed sets in both JCPyV- and BKPyV-infected cells compared to uninfected cells. HRPTE cells were infected with either JCPyV or BKPyV, and at 3, 6, and 9 dpi, total RNA was harvested and RNA-seq and bioinformatics analysis were performed. Genes from JCPyV- and BKPyV-infected cells were compared to those from uninfected cells. The lists of genes were uploaded to a gene enrichment analysis tool called Enrichr. Enrichr calculates *P* values using the Fisher exact test, and −log_10_ values (*P* values) are displayed for the top 9 biological pathways at 3, 6, and 9 dpi for JCPyV (A, C, and E) and for BKPyV (B, D, and F). Download Figure S1, EPS file, 1.6 MB

Table S1  BKPyV-infected cells overexpressed only four ISGs at 6 dpi. HRPTE cells were infected with either JCPyV or BKPyV, and at 3, 6, and 9 dpi, total RNA was harvested and RNA-seq and bioinformatics analyses were performed. Genes from JCPyV- and BKPyV-infected cells were compared to those from uninfected cells. Log_2_ fold change (FC) values are reported for the four ISGs found significantly overexpressed in BKPyV-infected cells. As a comparison, the log_2_ fold change values of the same four genes in JCPyV-infected cells are reported. Table S1, EPS file, 0.7 MB
