# Correction for Assetta et al., “JC Polyomavirus Infection of Primary Human Renal Epithelial Cells Is Controlled by a Type I IFN-Induced Response”

**DOI:** 10.1128/mBio.02354-19

**Published:** 2019-10-15

**Authors:** Benedetta Assetta, Marco De Cecco, Bethany O’Hara, Walter J. Atwood

**Affiliations:** a Graduate Program in Pathobiology, Brown University, Providence, Rhode Island, USA; b Department of Molecular Biology, Cell Biology, and Biochemistry, Brown University, Providence, Rhode Island, USA

## AUTHOR CORRECTION

Volume 7, issue 4, e00903-16, 2016, https://doi.org/10.1128/mBio.00903-16. RNA-seq data presented in the article have been made publicly available on the NCBI Gene Expression Omnibus site (GEO), accession number GSE135833.

